# Postoperative delirium in patients undergoing TAVI versus SAVR – A systematic review and meta-analysis

**DOI:** 10.1016/j.ijcha.2024.101544

**Published:** 2024-10-31

**Authors:** Dimitrios Stavridis, Angelique Runkel, Anna Starvridou, Johannes Fischer, Luca Fazzini, Hristo Kirov, Max Wacker, Jens Wippermann, Torsten Doenst, Tulio Caldonazo

**Affiliations:** a Department of Cardiothoracic Surgery, University Clinic Magdeburg, Magdeburg, Germany; b Department of Cardiothoracic Surgery, Jena University Hospital, Friedrich-Schiller-University Jena, Germany; c European University Cyprus, School of Medicine, Nicosia, Cyprus; d Department of Medical Sciences and Public Health, Clinical Cardiology Unit, University of Cagliari, Cagliari, Italy; e Department of Cardiovascular Medicine, Mayo Clinic, Rochester, MN, United States; f Department of Cardiothoracic Surgery, Weill Medical College of Cornell University, New York, United States

**Keywords:** Postoperative delirium, TAVI, SAVR, Meta-analysis

## Abstract

**Background:**

Transcatheter Aortic Valve Implantation (TAVI) and Surgical Aortic Valve Replacement (SAVR) have different levels of invasiveness which can result in different levels of functional status after the procedure.

**Methods:**

We performed a systematic review and meta-analysis to detect studies showing direct comparison between TAVI and SAVR regarding postoperative functional status. The primary endpoint was the incidence of postoperative delirium (POD) after TAVI or SAVR, assessed using the Confusion Assessment Method (CAM). Secondary endpoints included 30-day mortality, stroke, major bleeding, and hospital length of stay (LOS).

**Results:**

We identified 1,161 manuscripts, of which 10 studies (12,015 patients) were analyzed. TAVI patients had a significantly lower incidence of POD (OR: 0.35, 95 % CI, 0.26–0.48, p < 0.01) compared to SAVR patients. No significant differences were found in secondary outcomes between the groups.

**Conclusions:**

TAVI is associated with a lower incidence of postoperative delirium compared to SAVR without compromising length of stay or other major clinical outcomes. Further research is needed to understand the impact of postoperative delirium on short and long-term outcomes.

## 1 Introduction

Physical and cognitive performance following cardiac procedures is an important decision factor for heart teams in the process of choosing the best treatment plan for patients.

Transcatheter Aortic Valve Implantation (TAVI) and Surgical Aortic Valve Replacement (SAVR) have different levels of invasiveness which can result in different levels of functional status after the procedure. Postoperative delirium (POD) is a common and serious complication following both TAVI and SAVR [1]. Delirium, which is characterized by acute confusion and fluctuating levels of consciousness, can lead to prolonged hospital stays, increased healthcare costs, and higher morbidity and mortality rates [2]. Risk factors include advanced age, preexisting cognitive impairment, multiple comorbidities, and complex postoperative courses [3]. Early recognition and management of delirium are crucial for improving outcomes and enhancing recovery in these patients.

Greater invasiveness and longer narcosis time may be related to slower postprocedural mobilization and higher incidences of POD [4]. We therefore performed a systematic review and *meta*-analysis to detect studies showing direct comparison between TAVI and SAVR regarding postoperative functional status.

## 2 Methods

Ethical approval of this analysis was not required as no human or animal subjects were involved. This review was registered with the National Institute for Health Research International Registry of Systematic Reviews (CRD42023451208).

We performed a comprehensive literature search according to the Preferred Reporting Items for Systematic Reviews and Meta-Analyses (PRISMA) guidelines [5]. MEDLINE, Embase and Google Scholar were searched for the terms: “aortic valve replacement” AND (“functional status” OR “physical performance” OR “delirium”). After de-duplication, the records were screened by two independent reviewers (DM and AR). Any discrepancies and disagreements were resolved by a third author (TC).

The primary endpoint was the incidence of postoperative delirium after TAVI or SAVR, using the Confusion Assessment Method (CAM) [6] and secondary endpoints included 30-day mortality, stroke, major bleeding, and hospital length of stay (LOS).

Odds ratios (OR) with 95% confidence interval (CI) and p-values were calculated for each of the clinical outcomes. Standard mean difference (SMD) was calculated for the continuous variables. Chi-squared and I^2^ tests were executed for the assessment of statistical heterogeneity [7]. By using a random-effects model, the ORs were combined across the studies [8]. All analyses were completed through the “metafor” package of R Statistical Software (version 4.0.2), Foundation for Statistical Computing, Vienna, Austria.

## 3 Results

The search revealed 5,112 articles, of which, 1,161 were screened and 10 studies (Table 1), with overall 12,015 patients (2,843 TAVI and 9,172 SAVR patients), were included in the final analysis [9], [10], [11], [12], [13], [14], [15], [16], [17], [18].

**Table 1:** Summary of the included studies.

Author	Year of Publication	Country	N° of patients (TAVI/SAVR)	Study Design	Risk Adjustment
Hoogma et. al.	2003	Belgium	250 (84/166)	P, NR, SC	PSM
Humbert et. al.	2021	Switzerland	93 (66/27)	P, NR, SC	None
Bo et. al.	2020	Italy	154 (109/45)	P, NR, SC	MVR
Shi et. al.	2019	United States	187 (110/77)	P, NR, SC	None
Kim et. al.	2019	United States	246 (143/103)	P, NR, SC	None
Eggebrecht et. al.	2018	Germany	6972 (1268/5708)	R, MC	PSM
Maniar et. al.	2016	United States	427 (168/259)	R, SC	PSM
Eide et. al.	2016	Norway	136 (63/73)	P, NR, SC	MVR
Eide et. al.	2015	Norway	143 (65/78)	P, NR, SC	None
Bestehorn et. al.	2015	Germany	3407 (771/2636)	R, MC	PSM

MC  = multicenter, MVR  = multivariate regression, NR  = non-randomized, P  = prospective, PSM  = propensity score matching, R  = retrospective, SAVR  = surgical aortic valve replacement, SC  = single center, TAVI = transcatheter aortic valve implantation.

**Fig. 1:**
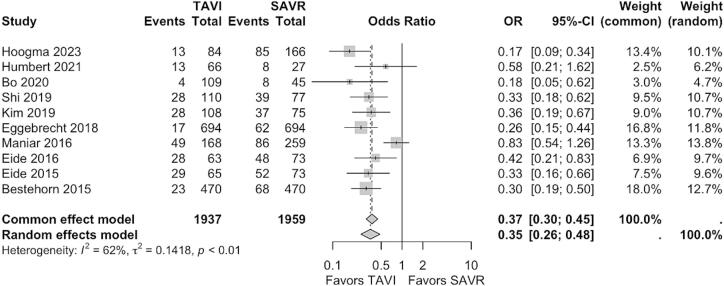
Forest plot for post operative delirium. CI: confidence interval, OR: odds ratio, SAVR: surgical aortic valve replacement, TAVI: transcatheter aortic valve implantation.

Fig. 1 shows the forest plot of the primary outcome. Patients after TAVI demonstrated significantly lower incidence of POD (OR: 0.35, 95% CI, 0.26–0.48, p<0.0001).

**Fig. 2:**
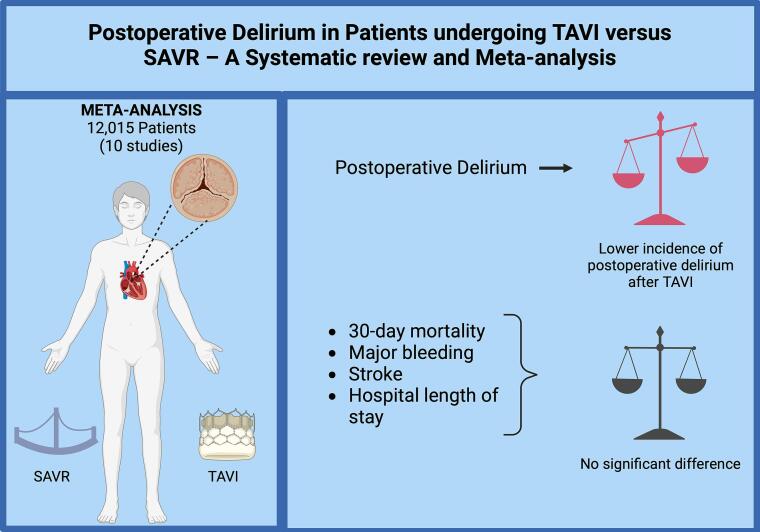
Graphical abstract. SAVR: surgical aortic valve replacement, TAVI: transcatheter aortic valve implantation.

Table 2 and Fig 2. summarize the main findings of this analysis. Regarding the secondary outcomes, there was no statistically significant difference between the TAVI and the SAVR group regarding the 30-day mortality (OR: 0.71, 95% CI, 0.35–2.86, p  = 0.99), stroke (OR: 1.25, 95% CI, 0.59–2.631, p = 0.56), major bleeding (OR: 1.09, 95% CI, 0.15–8.19, p = 0.93), and hospital LOS (SMD: −2.09, 95% CI, −4.93 to 0.74, p = 0.15).

**Table 2:** Outcomes summary.

Outcome	Number of studies	Number of patients	Effect estimate (95 % confidence interval, p-value)
Postoperative delirium	10	3,896	OR: 0.35, 95 % CI, 0.26–0.48, p < 0.0001
30-day mortality	4	2,609	OR: 0.71, 95 % CI, 0.35–2.86, p = 0.99
Stroke	5	2,460	OR: 1.25, 95 % CI, 0.59–2.631, p = 0.56
Major bleeding	4	2,033	OR: 1.09, 95 % CI, 0.15–8.19, p = 0.93
Hospital LOS	4	2,219	SMD: −2.09, 95 % CI, −4.93 to 0.74, p = 0.15

CI: confidence interval, LOS: length of stay, OR: odds ratio, SMD: standard mean difference.

Only two studies reported objective outcomes regarding the functional status of the patients, such as the Mini Mental Status Examination (MMSE), the clock drawing test or the MOCA questionnaire. Nonetheless, data was not available from all studies and therefore deemed as insufficient to be included in the *meta*-analysis.

## 4 Discussion

Our analysis demonstrates that the incidence of POD is lower among TAVI patients in comparison with SAVR. This can be attributed to some factors, which are already expected. First and foremost, it is standard practice among many heart teams, that TAVI shall be performed under mild sedation, in comparison to SAVR, where general anesthesia is required [19]. Secondly, SAVR is directly related to the use of cardiopulmonary bypass, which may be related to a greater inflammatory response in the postoperative period [20].

However, the higher incidence of POD in the SAVR cohort does not correlate with hospital LOS or mortality. This could implicate that delirium is either not as relevant in SAVR, or that delirium is not a causal factor, but only a surrogate for identifying the sicker patients. In this context, POD may occur more frequently in SAVR, perhaps because the “stress” for the patient is different.

Although TAVI is generally associated with improved short-term outcomes, such as reduced mortality and stroke rates, our findings did not reflect these advantages, even in a large patient cohort. One potential explanation, as discussed in the literature, could be the variability in patient populations, procedural techniques, and follow-up protocols across different studies. Additionally, factors such as the severity of comorbidities, the timing of intervention, and the skill level of the surgical teams may influence outcomes. Further investigation into these variables is necessary to understand the observed discrepancies fully.

The current literature on this topic reports the incidence of POD, with minimal information regarding the severity or duration of the delirium or the physical and cognitive performance after both procedures [9], [10], [11], [12], [13], [14], [15], [17]. While TAVI patients present a significantly lower incidence of POD, there is no evidence if and how POD is associated with a decline in functional status and physical performance, both short- and long-term [9], [10], [11], [12], [14], [15], [16], [17], [18]. This raises also the question whether POD could be associated with the incidence of major cardiovascular and neurological complications in the long run. Prospective data and randomized controlled trials are needed to shed light into this topic and further equip the heart teams with evidence-based data for the optimal clinical decision.

## CRediT authorship contribution statement

**Dimitrios Stavridis:** Writing – original draft, Methodology, Data curation, Conceptualization. **Angelique Runkel:** Writing – original draft, Investigation, Formal analysis, Data curation, Conceptualization. **Anna Starvridou:** Writing – original draft, Visualization. **Johannes Fischer:** Writing – review & editing, Visualization, Validation, Supervision. **Luca Fazzini:** Writing – review & editing, Validation, Methodology. **Hristo Kirov:** Writing – review & editing, Visualization, Validation, Supervision. **Max Wacker:** Visualization, Validation, Supervision, Conceptualization. **Jens Wippermann:** Writing – review & editing, Visualization, Validation, Supervision. **Torsten Doenst:** Visualization, Validation, Supervision. **Tulio Caldonazo:** Writing – original draft, Formal analysis, Data curation, Conceptualization.

## Declaration of competing interest

The authors declare that they have no known competing financial interests or personal relationships that could have appeared to influence the work reported in this paper.
